# Massive Hepatomegaly in a Pediatric Patient Due to a Solitary Simple Hepatic Cyst: A Case Report

**DOI:** 10.7759/cureus.70249

**Published:** 2024-09-26

**Authors:** Zahra A Hasan

**Affiliations:** 1 College of Medicine, National University of Science and Technology, Muscat, OMN

**Keywords:** case report, conservative management, hepatomegaly, imaging, pediatric, simple hepatic cyst

## Abstract

This case report presents a six-year-old male patient with massive hepatomegaly attributed to a solitary simple hepatic cyst. The patient was referred to the pediatric gastroenterology clinic after his family noted progressive abdominal distention over four months, with no associated symptoms such as pain, jaundice, or systemic illness. Physical examination revealed significant hepatomegaly and initial laboratory investigations indicated mild transaminitis but were otherwise unremarkable. Imaging with abdominal ultrasound demonstrated a single large cystic lesion, which was further characterized by computed tomography (CT) as a 12-cm simple hepatic cyst. The differential diagnosis included conditions such as polycystic liver disease and hydatid disease, but these were ruled out based on clinical presentation and imaging findings. Given the benign nature of the cyst and the absence of symptoms, conservative management was pursued, with regular follow-up to monitor for potential complications. The patient remained asymptomatic during a six-month follow-up, highlighting the importance of individualized monitoring and management in cases of pediatric hepatic cysts. This case contributes to the understanding of simple hepatic cysts in children and underscores the need for careful diagnostic evaluation and long-term follow-up.

## Introduction

Simple hepatic cysts are fluid-filled cavities within the liver, generally considered benign and asymptomatic. They are a rare finding in the pediatric population and are more commonly identified in adults, in whom they often remain clinically silent. In children, hepatic cysts may be discovered incidentally during imaging for unrelated reasons or when they reach a significant size, leading to symptoms like abdominal distention, discomfort, or hepatomegaly [1]. The etiology of simple hepatic cysts remains largely unknown, although they are thought to arise from developmental malformations of the bile ducts. Unlike polycystic liver disease or hydatid cysts, which may be associated with genetic or infectious causes, simple hepatic cysts are isolated lesions with no known familial or systemic associations [1,2].

Massive hepatomegaly in pediatric patients can be a challenging clinical scenario, as it may be a manifestation of various underlying conditions, ranging from infectious, metabolic, neoplastic, or congenital disorders. In particular, hepatic cystic lesions prompt consideration of several differential diagnoses, including polycystic liver disease, cystic tumors such as biliary cystadenoma, hydatid disease caused by *Echinococcus* infection, or biliary atresia with cystic degeneration [2,3]. The diagnostic challenge lies in differentiating between these potentially serious conditions and simple hepatic cysts, which have a benign course and often do not require intervention. Diagnostic imaging plays a crucial role in determining the nature of hepatic cysts, with ultrasound commonly being the first-line modality. Computed tomography (CT) and magnetic resonance imaging (MRI) provide further anatomical details, helping to exclude malignant or infectious processes [4].

In most pediatric cases, simple hepatic cysts remain asymptomatic and are managed conservatively. However, in cases where the cysts become large enough to cause mechanical compression on adjacent organs or induce symptoms such as pain or respiratory difficulty, more aggressive interventions like cyst aspiration, marsupialization, or surgical excision may be considered. Despite their size, these cysts rarely cause complications such as rupture, hemorrhage, or secondary infection. Regular follow-up with imaging is often recommended to monitor cyst size and identify any potential complications early [2,3].

This case report highlights a rare presentation of massive hepatomegaly in a pediatric patient caused by a solitary simple hepatic cyst. The rarity of such a presentation underscores the importance of a thorough diagnostic approach to hepatomegaly in children, and the necessity of distinguishing between benign cystic lesions and more serious pathologies that may warrant intervention. The case contributes valuable insight into the clinical course and management of large simple hepatic cysts in the pediatric population.

## Case presentation

A six-year-old previously healthy male was referred to our pediatric gastroenterology clinic due to progressive abdominal distention over the past four months. His mother reported noticing a gradual increase in the size of his abdomen, initially attributed to bloating. However, as the distention worsened, they sought medical attention. The child denied any associated pain, vomiting, diarrhea, or changes in bowel habits. His appetite had slightly decreased, though he was still able to tolerate a normal diet. There were no recent changes in weight, and the family denied any history of trauma, fever, or systemic symptoms such as jaundice or fatigue. His past medical history was unremarkable, with no history of liver disease, hospitalizations, or surgeries. The family history was negative for liver disease, malignancies, or genetic disorders. The patient’s developmental milestones were appropriate for his age.

On physical examination, the patient appeared well-nourished and in no acute distress. His vital signs were within normal limits for his age. Upon inspection, the abdomen was distended but non-tender. There was no visible abdominal skin discoloration, venous dilation, or ascites. Palpation revealed a firm, non-painful, massive hepatomegaly, with the liver extending several centimeters below the costal margin. No splenomegaly was appreciated. Bowel sounds were present and normal, and there were no signs of peritonitis or palpable masses. The remainder of his systemic examination, including cardiovascular, respiratory, and neurological assessments, was unremarkable.

Initial laboratory investigations included a complete blood count, which revealed no abnormalities. Liver function tests showed mildly elevated alanine aminotransferase (ALT) at 58 U/L (normal <40 U/L) and aspartate aminotransferase (AST) at 64 U/L (normal <40 U/L). Total bilirubin and direct bilirubin levels were within normal limits, as were alkaline phosphatase, gamma-glutamyl transferase (GGT), and albumin. Coagulation studies, including prothrombin time (PT) and international normalized ratio (INR), revealed normal readings. Serologic testing for viral hepatitis (hepatitis A, B, and C), Epstein-Barr virus, and cytomegalovirus was negative. An autoimmune hepatitis panel, including antinuclear antibodies (ANA), anti-smooth muscle antibodies (ASMA), and liver-kidney microsomal antibodies (LKM), was also negative.

**Figure 1:**
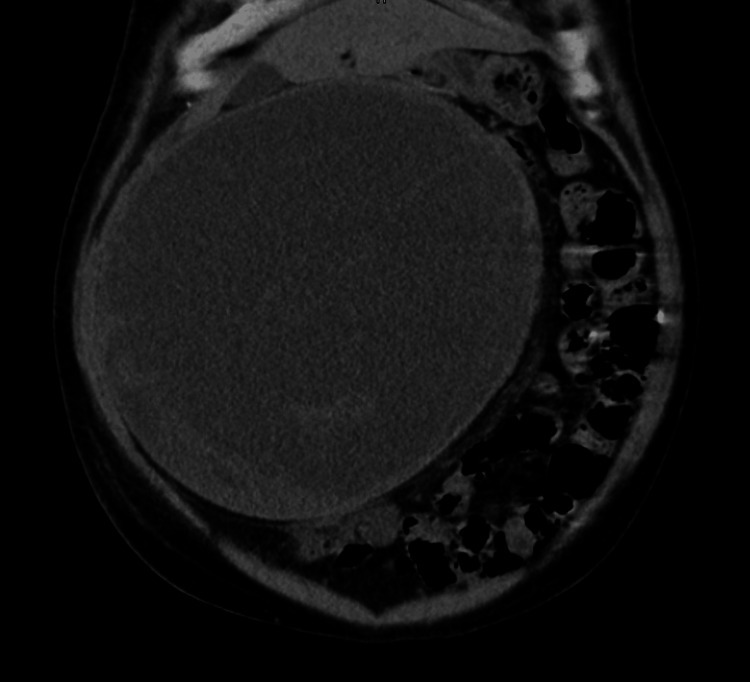
Coronal CT Image of the abdomen Coronal CT image of the abdomen depicting a large cystic lesion originating from the inferior border of the liver. The cyst features thin enhancing septations without any solid components or calcifications. There is no evidence of intrahepatic biliary dilation, and no other hepatic lesions are present. The mass exerts a significant mass effect on the adjacent bowel loops, indicating potential displacement.

Given the significant hepatomegaly and unremarkable initial lab workup, imaging was pursued. Abdominal ultrasound revealed a single large cystic lesion within the liver, measuring 12 cm in diameter. The cyst was anechoic, with well-defined margins and no internal septations, consistent with a simple hepatic cyst. Doppler studies showed normal hepatic blood flow and there was no evidence of portal hypertension. To further characterize the cyst and rule out other possible etiologies, a computed tomography (CT) scan of the abdomen was performed. The CT confirmed the presence of a solitary, non-septated hepatic cyst with no solid components, calcifications, or evidence of malignancy. There was no involvement of other abdominal organs, and the biliary tree appeared normal. These findings were consistent with a diagnosis of a simple hepatic cyst (Figure 1).

The differential diagnosis for massive hepatomegaly with a cystic lesion in a pediatric patient includes conditions such as polycystic liver disease, hydatid disease, biliary cystadenoma, and cystic neoplasms. However, the absence of multiple cysts, systemic symptoms, and characteristic imaging findings excluded these possibilities. Based on the clinical presentation and imaging, a diagnosis of massive hepatomegaly secondary to a simple hepatic cyst was made.

The patient’s management was conservative given the benign nature of the cyst and the absence of symptoms directly related to the cyst, such as pain, infection, or biliary obstruction. Surgical intervention or cyst drainage was not indicated as the cyst was asymptomatic, and there was no evidence of complications such as rupture, hemorrhage, or secondary infection. The family was counseled on the benign course of simple hepatic cysts, and the patient was scheduled for regular follow-up to monitor the size of the cyst and potential complications. Nutritional support and lifestyle modifications were discussed to ensure optimal growth and development despite the hepatomegaly.

During follow-up at six months, the patient remained asymptomatic, with no further increase in abdominal distention. Repeat ultrasound showed stable cyst size with no new complications. Liver function tests remained within normal limits, and the patient continued to thrive, attending school and participating in normal activities. A long-term follow-up plan was established to monitor for any changes in cyst size or the development of symptoms.

## Discussion

The presentation of massive hepatomegaly due to a solitary simple hepatic cyst in a pediatric patient, as described in this case, is rare and presents unique diagnostic and management challenges. Simple hepatic cysts, while common in adults, are infrequently encountered in children, particularly when they reach a size that results in significant hepatomegaly [2-5]. This discussion aims to highlight the clinical implications of such a presentation, examine the differential diagnosis, review the current literature on management strategies, and emphasize the importance of long-term follow-up in pediatric patients with hepatic cysts.

Simple hepatic cysts are generally considered congenital lesions resulting from the malformation of bile duct structures during embryogenesis. While most hepatic cysts remain asymptomatic and undetected throughout life, large cysts can present with symptoms related to mass effect, including abdominal distention, discomfort, and rarely, complications such as cyst rupture, hemorrhage, or infection [1,2]. The literature on simple hepatic cysts in children is sparse, with most data are derived from adult studies. In children, the discovery of a large hepatic cyst often raises concerns of more sinister causes, including hydatid cysts, biliary cystadenomas, or cystic liver neoplasms, which must be ruled out through careful imaging and laboratory evaluation [4-6].

The differential diagnosis for a large cystic lesion in the liver of a pediatric patient is broad and includes a variety of benign, infectious, and malignant entities. Polycystic liver disease, though rare in isolation, can be part of an autosomal dominant polycystic kidney disease spectrum. Hydatid disease, caused by *Echinococcus* infection, must be considered, especially in patients from endemic regions or with a history of animal exposure [1,6]. Biliary cystadenomas are rare, potentially malignant neoplasms that also present as large, solitary cystic masses and can be challenging to differentiate from simple cysts based on imaging alone. In our case, the absence of systemic symptoms, negative serology for infectious causes, and characteristic imaging findings helped to establish the diagnosis of a simple hepatic cyst and exclude other causes [3,5].

In terms of management, simple hepatic cysts are typically benign and asymptomatic, with observation being the standard of care in most cases. Surgical intervention is reserved for cysts that cause symptoms due to compression of adjacent organs or complications such as rupture or infection [2,3]. In this case, despite the massive size of the cyst, the patient remained asymptomatic, and conservative management was chosen. The decision to avoid intervention was supported by the absence of clinical symptoms or imaging findings suggestive of imminent complications. However, the potential for future complications, including cyst growth or secondary infection, necessitates regular follow-up with serial imaging to monitor the cyst’s size and any associated changes.

The literature on the long-term outcomes of pediatric patients with simple hepatic cysts is limited [4-6]. In most cases, cysts remain stable or grow slowly over time, with only a small fraction of patients requiring intervention. Nonetheless, the size of the cyst in this case, coupled with the degree of hepatomegaly, underscores the importance of long-term monitoring. While the risk of malignancy is exceedingly low in simple cysts, continued vigilance is essential, particularly as the child grows, to ensure that any changes in the cyst’s characteristics or the emergence of symptoms are promptly addressed.

This case also highlights the diagnostic utility of imaging in distinguishing simple hepatic cysts from more complex or malignant cystic lesions. Abdominal ultrasound is often the first-line imaging modality, offering a non-invasive and readily available method for identifying cystic lesions. However, ultrasound findings alone may not be sufficient to exclude other differential diagnoses, such as biliary cystadenoma or hydatid cysts, which may have overlapping sonographic features. In this case, a CT scan was performed to provide further anatomical detail, confirming the benign nature of the cyst. CT imaging plays a critical role in delineating cyst morphology, ruling out solid components, calcifications, or septations, and assessing the relationship of the cyst to adjacent structures. Magnetic resonance imaging (MRI) may also be useful, particularly in cases where there is diagnostic uncertainty or suspicion of a more complex cystic lesion.

## Conclusions

In summary, this case of massive hepatomegaly due to a solitary simple hepatic cyst in a pediatric patient emphasizes the importance of a thorough diagnostic approach, including detailed imaging and clinical evaluation, to distinguish benign cysts from other potentially serious conditions. Despite its large size, the cyst remained asymptomatic, allowing for conservative management with regular follow-up, underscoring the generally benign nature of simple hepatic cysts in children. This case highlights the need for continued vigilance in monitoring these lesions, even when asymptomatic, to promptly address any complications that may arise. The rarity of such presentations in pediatrics adds valuable insight to the literature and reinforces the importance of individualized care in managing cystic liver lesions.
